# Intergenerational effects of dietary changes on trait means and variance

**DOI:** 10.1098/rsbl.2025.0310

**Published:** 2025-09-24

**Authors:** Marcus Lee, Kevin Tran, Yasmine Castillo, Matthew R. Walsh

**Affiliations:** ^1^ Functional Ecology, Department of Biology, Lund University, Lund, Sweden; ^2^ Department of Biology, The University of Texas at Arlington, Arlington, TX, USA

**Keywords:** maternal effects, trait variability, developmental plasticity, environmental canalization, zooplankton

## Abstract

Environmental stress can alter not only trait means but also trait variances—an often-overlooked evolvable feature with ecological and evolutionary relevance. We examine how dietary stress affects both the mean and variance of morphological and reproductive traits across two generations in clonal *Daphnia*. We manipulated maternal and offspring environments with high- (algae) or low-quality (cyanobacteria) diets, measuring eye size, body size and reproductive output in eight genotypes. Morphological trait means showed consistent treatment-by-generation interactions: low-quality diets reduced trait size, with partial recovery upon re-exposure to high-quality food. Reproduction was largely determined by current conditions, while eye and body size showed legacy effects of maternal environment. Variance patterns were trait-specific: eye size variance declined under stress, body size variance increased across generations and reproductive variance peaked in offspring released from maternal stress. We developed a conceptual framework considering roles for condition transfer, anticipatory plasticity and diversified bet-hedging as potential mechanisms underlying these intergenerational impacts on trait variances. Although no single mechanism explained all outcomes, our findings tentatively support condition transfer and suggest potential co-occurrence of multiple strategies. This study underscores the importance of jointly examining mean and variance responses to stress to better understand phenotypic plasticity and its evolutionary consequences.

## 1. Introduction

Variation is a fundamental aspect of evolution. Specifically, heritable differences among individuals within a population serve as the raw material upon which natural selection acts. However, phenotypic variation is not strictly intrinsic; rather, it emerges from the confluence of genetic expression, physiological processes and ecological heterogeneity [1–4]. Selection will favour individuals that maximize fitness under such environmental perturbations. Consequently, various strategies have evolved that mediate the effects of selection by altering trait variation, such as phenotypic plasticity [2] and transgenerational or intergenerational mechanisms like bet-hedging [5,6].

Despite long-standing interest in trait variation, most empirical work has focused on central tendency measures (mean, median, etc.) to understand phenotypic responses [7]. Yet , trait variance is increasingly recognized as a biologically meaningful feature independent of the mean, with effects across multiple levels of biological organization [8,9,10,11]. Moreover, trait variance itself has been shown to be evolvable [12–14], further underscoring its potential evolutionary significance [15].

A key challenge in studying how variance responds to environmental stress is the lack of consistent theoretical predictions for the direction of change. In a simplified scenario of two contrasting environments—one benign and one stressful—some studies suggest that phenotypic variance increases under stress [16,17]. This may result from heightened genetic variation under directional selection or the expression of cryptic variation that remains unexpressed under neutral conditions. If we also assume intrinsic variation exists, stress may exacerbate it through developmental instability, producing greater among-individual trait differences [18,19]. Conversely, intense stress could reduce trait variance via strong selection against suboptimal phenotypes [16]. Similarly, Oleksiak & Crawford [20] propose that physiological responses to stress may reduce among-individual trait variance. According to this hypothesis, the physiological imperative to maintain homeostasis drives individuals toward a similar stressed phenotype, effectively reducing variance—a process termed environmental canalization [21]. These contrasting predictions highlight how assumptions about the nature and intensity of environmental stress directly shape expectations for trait variance.

Among stress-response strategies, within-generation plasticity is the most widely studied. Defined as the ability of a single genotype to express multiple phenotypes, plasticity is often considered adaptive [22], though non-adaptive or maladaptive plasticity can also occur [23]. Yet, mean and variance outcomes are not straightforward to predict. From an adaptive standpoint, if a particular mean phenotype confers a fitness advantage under stress, then trait variance is expected to be low. Adding further complexity, the effects of prior generations on present-day phenotypes are well documented [5,24–28]. Frameworks such as condition-transfer effects (i.e. the transmission of the parental condition to the offspring [29]) or anticipatory intergenerational/transgenerational plasticity (i.e. the priming of offspring/grand-offspring by the parent for the anticipated environment experienced by the offspring [5,25]) seek to account for such cross-generational influences. These frameworks primarily focus on the mean, however, and they often do not make explicit predictions about trait variance. In contrast, bet-hedging theory predicts that parents may increase trait variance among offspring to reduce variance in genotype-level fitness [6,30,31]. While plasticity and bet-hedging are often framed as distinct strategies, recent work suggests they likely co-occur [32–34]. This further blurs the expectations for the outcome of trait variance.

Clonal organisms such as *Daphnia* spp. offer the opportunity to test the influence of stress on trait means and variance. Parthenogenetic organisms enable researchers to maintain multiple genotypically distinct lines and replicate individuals within each genotype, facilitating analyses at the genotypic or individual level [35]. *Daphnia* are also a well-established laboratory model, with extensive documentation of genotype-by-environment interactions [3,36], transgenerational effects [37–41] and bet-hedging strategies [42,43]. Furthermore, dietary variation is well studied in *Daphnia*, ranging from the ingestion of nutritionally deficient algae to morphologically inedible or toxin producing cyanobacteria [37,44–46]. One particularly salient feature of cyanobacteria is the deficiency of essential polyunsaturated fatty acids, which can constrain reproductive success [44]. This makes dietary stress—commonly explored via contrasting high-quality (algae) and low-quality (cyanobacteria) diets [47]—a tractable model system to investigate both within- and across-generation plasticity. This is in part due to the strong link between maternal condition on offspring size through energetic provisioning [48]. However, studies that jointly examine changes in trait means and variances across generations remain rare [49].

Here, we test how the influence of diet quality shapes the mean and variance of morphological and reproductive traits of *Daphnia*. We exposed maternal and offspring generations to either a ‘high-’ or ‘low-’quality diet, with an additional group in which the maternal generation experienced ‘low-quality’ food, while their offspring received ‘high-quality’ food. Our general hypothesis is that dietary stress affects both the mean and variance of each trait.

**Figure 1.**
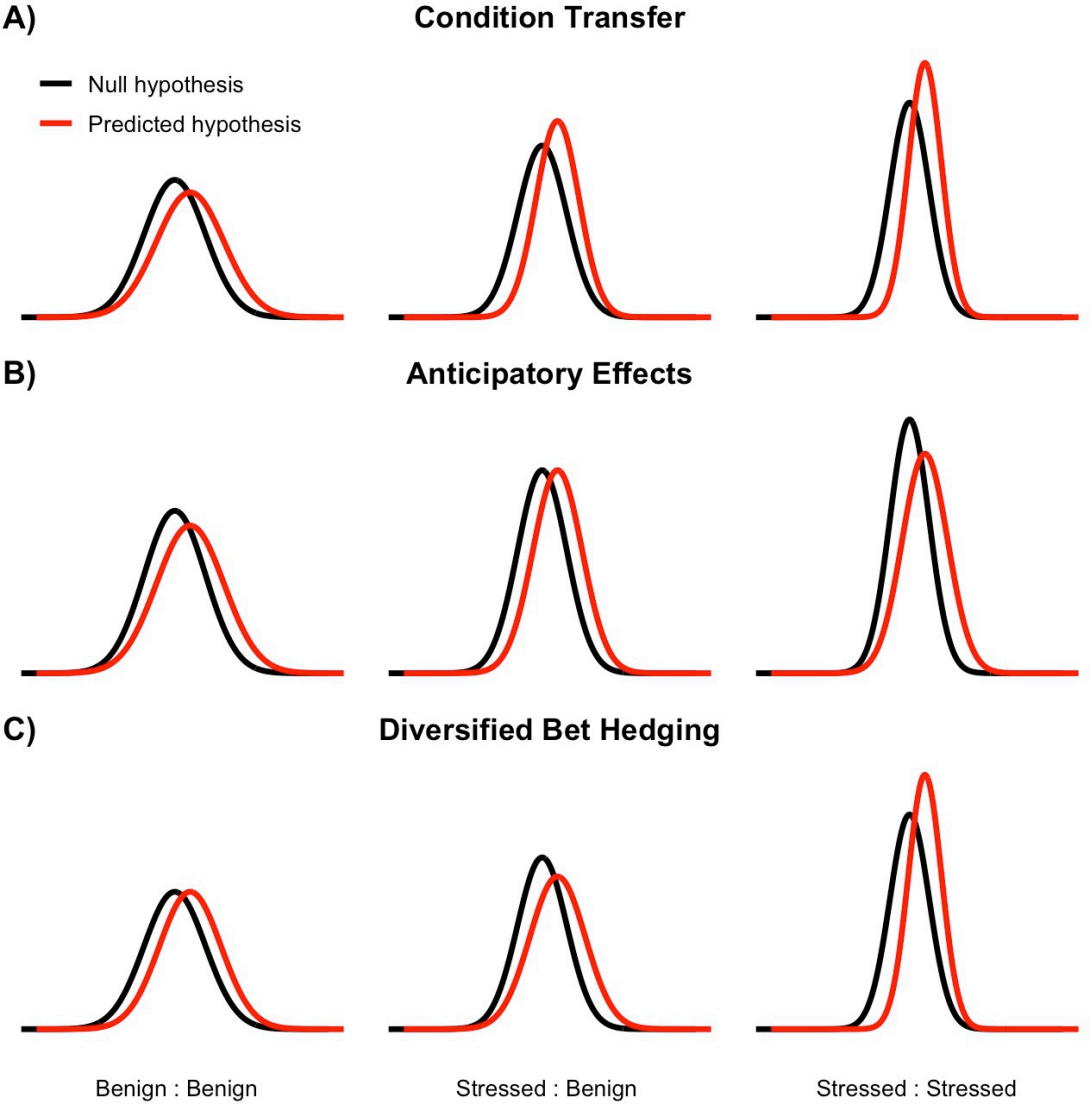
Hypothetical distributions of trait variance under three potential cross-generational mechanisms; condition transfer (A), anticipatory effects (B) and diversified bet-hedging (C) strategies. Baseline variation was set at 1 and stress response at 0.2. The red line is the expected variance according to equation (1). The black line is the null hypothesis that an equally weighted interaction between maternal and offspring environment determines variance (box 1). Each distribution was calculated with the same mean, but adjusted on the *x*-axis by an increment of 0.25 to prevent overlapping distributions for visibility. The environmental conditions are written along the *x*-axis (parental environment : offspring environment). Under these conditions, no transfer mechanism displays the same distribution as another mechanism for all of the proposed experimental combinations.

Based on similar prior work [47], we predicted that mothers exposed to ‘low-quality’ diets would exhibit decreased means for all traits in both the parental and offspring generations. We also predicted a decrease in within-generation variance, consistent with the view of plasticity and physiological stress responses as forms of environmental canalization [20,21]. For the offspring generation, we explicitly considered three potential mechanisms of cross-generational transfer such as condition transfer [28], anticipatory effects [25] and bet-hedging [31]. Under condition transfer effects, we predict offspring trait means to be higher when mothers are fed high-quality food. If anticipatory effects dominate, we predict that individuals matched to their maternal environment would have higher trait means. However, if bet-hedging is the primary mechanism, we expect a lack of a mean response, but an increase in variance, observable in the maternally mismatched environment (figure 1). Further to the basic expectations of the mean, we constructed a simplified framework of variance expectations for these three cross-generational mechanisms (table 1; box 1). This study then provides a rare empirical test of how trait-specific plasticity and cross-generational responses jointly shape both trait means and variances under dietary stress, thereby offering new insights into the eco-evolutionary dynamics of phenotypic variation.

Box. 1 **Conceptual framework for intergenerational stress effects on variance.**

Understanding how intergenerational consequences emerge from stress requires testable hypotheses for both the mean and the variance. While much work and attention have been placed upon the mean, it is far less clear how variance is affected by prior generations [9,14]. Furthermore, there are multiple potential non-mutually exclusive cross-generational strategies, which either confound the expectations or do not make clear any specific effects on variance [25,28–30]. Broadly speaking, these can be classified into three primary categories such as ‘condition transfer’, ‘anticipatory’ and ‘bet-hedging’. Condition transfer suggests the quality or resource availability of the parents will determine the offspring phenotypes [28,29]. Anticipatory effects are widely expected but not readily observed and imply that if the parent’s environment predicts the offspring environment, then the offspring will have higher fitness than those that did not predict the coming environment [50,51]. Conversely, bet-hedging is predicted to emerge when the autocorrelation of the parents’ and offspring’s environment is low [6,30,31]. There are specific expectations regarding variance for bet-hedging strategies, but the other strategies only contain the implicit assumptions depending on the type of data, such as the mean equalling the variance in the case of a Poisson distribution [52]. As a result, we propose that a conceptual framework of mean-independent variance expectations in the hopes to provide a greater understanding of the intergenerational strategies that underlie genotypic fitness (equation 1).

$$\sigma_{M,i}^{2}=\left\{ \begin{array}{l} E_{P,i}\cdot E_{C,i}+\frac{s}{2},\;\;M_{i}=1\\ E_{P,i}\cdot E_{C,i},\;\;\;\;\;\;\;\;\;\;M_{i}=0\\ E_{P,i}\cdot E_{C,i}-\frac{s}{2},\;\;M_{i}=-1 \end{array}\right.$$

Equation 1 defines the adjusted variance (*σ^2^*_*M*,*i*_) for each condition (*i*), based on the product of the parental environment effect (*E*_P,i_) and current environment effect (*E*_C,i_), modulated by the mechanism type (*M*_*i*_). When *M*_*i*_ = 1, representing positive variance modulation, an additive shift of + $\frac{s}{2}$ is applied, where (s) denotes the within-generation stress response. When *M*_*i*_ = −1, indicating negative modulation, a subtractive shift of – $\frac{s}{2}$ is applied. If *M*_*i*_ = 0, no adjustment is made to the base variance.

Predictions regarding each of the three mechanisms (*M*_condition_, *M*_anticipatory_ and *M*_bet-hedging_), under each of the parental × current environment conditions, yield one of the three outcomes such as negative (−1), neutral (0) or positive (1). Condition transfer predicts positive effects for offspring from mothers in a benign environment, while negative effects if from a ‘stressed’ mother. Following the predictions regarding the mean under anticipatory effects, we suggest that variance will respond positively in predictable environments (either benign to benign or stressed to stressed). This implies that environments that are unpredictable garner negative responses; however, if the environment goes from stressed to benign, there is a lack of selection so we determined this specific example would result in a neutral response. In contrast, bet-hedging (specifically ‘diversifying’ bet-hedging) should produce higher variance in unpredictable environments. This would suggest that under consistently stressful environments bet-hedging would result in a negative response; however in a consistently benign environment, it would not confer either advantage or disadvantage.

We assume that stress (s) reduces within-generation phenotypic variance (*σ^2^*) of the sub-population [20,21] and that this response is consistent across generations i.e. the difference in variance between a population in a benign environment and a stressed one will be the same as a population that is in a benign environment, but their offspring are in a stressful environment. Therefore, under a fully factorial design, it would be possible to examine how stress effects are transferred across generations under each parental–offspring environment combination (electronic supplementary material, figure S1). We propose multiple null hypotheses; (i) variance is determined entirely by the current conditions (*E*_C_), (ii) variance is predominantly determined by the parental environment (*E*_P_) and (iii) variance is determined by the equal interaction of parental environment and current environment (*E*_P_*E*_C_). Our hypothesis would then be that variance is a product of intergenerational transfer strategy and the interaction between prior and current environmental conditions (figure 1).

**Table 1.** Predicted effects of intergenerational environmental conditions on trait means and variances in the offspring under three hypothesized maternal effects mechanisms. A ‘+’ or ‘−’ indicates a predicted increase or decrease relative to the previous generation, while ‘=’ indicates no expected change.

mechanism	intergenerational environment	mean	variance
condition transfer	benign : benign	=	+
	stressed : benign	+	—
	stressed: stressed	—	—
anticipatory effects	benign : benign	=	+
	stressed : benign	=	=
	stressed: stressed	+	+
diversified bet-hedging	benign : benign	=	=
	stressed : benign	=	+
	stressed: stressed	=	—

## 2. Methodology

### (a) Experimental design

*Daphnia pulicaria* and dietary cultures were maintained using established protocols (electronic supplementary material, S1 for all procedures). To minimize maternal effects of wild-caught to laboratory culture, a single individual per clonal line (*n* = 8; G0) was selected at random, from which two generations were produced under the same conditions, each from the second clutch. This second generation (G2) became the ‘first’ experimental generation. Approximately 15 individuals from each clone were placed into each treatment group such as AA, CA and CC. AA individuals were initially exposed to algae, and their daughters are also exposed to algae. CA individuals were first exposed to cyanobacteria, but their daughters were supplied with algae. CC individuals were exposed to cyanobacteria over both generations. This yielded an average sample size per treatment × generation group of 118.5 ± 1.5 s.d. (total *n* = 711 individuals). This experiment utilized *Tetradesmus obliquus* (UTEX 393) as the algae, which served as the ‘high-quality’ food and a non-toxic strain of cyanobacteria (*Anabaena inaequalis* UTEX 380) as the ‘low-quality’ food based on previously reported mean responses [47]. Under these conditions, we measured body size and eye size at 132 h ± 12 h old, as well as the reproductive output in the first three clutches of offspring. These measurements were made using ImageJ [53] on both generations and served as the dependent variables in the analyses.

### (b) Statistical analysis

All statistical analyses were performed using the software R v. 4.4.2 ‘Pile of Leaves’ [54]. To analyse the mean and variance differences of each trait, we utilized linear mixed models using the ‘lme’ function [55]. We entered ‘treatment’, ‘generation’ and their interaction as fixed effects and ‘age’ as a covariate for eye and body size. ‘Clone ID’ was included as a random factor. This function also permitted the specification of the residual variance (*σ*^2^) via the weights argument. Therefore, we compared the same base model as described above with four different variance structures such as constant variance, treatment-level variance, generation-level variance and the interaction of treatment- and generation-level variance. These models were then compared by likelihood ratio tests to examine the statistical significance of differing variance structures [49]. Our selection process involved prioritizing the model with the lowest Akaike information criterion (AIC) value. In cases where models did not significantly differ (*p* > 0.05), we preferred the simplest model structure. Using a linear mixed-effects model, we also calculated broad-sense heritability (*H*^2^) as the portion of variation attributable to the clonal ID while also considering genotype-by-environment interactions using the formula:


$$H^{2}=\frac{\sigma_{\text{clone}}^{2}+\sigma_{\text{clone}\times \text{environment}}^{2}}{\sigma_{\text{clone}}^{2}+\sigma_{\text{clone}\times \text{environment}}^{2}+\sigma_{\text{residual}}^{2}}$$

## 3. Results

**Figure 2.**
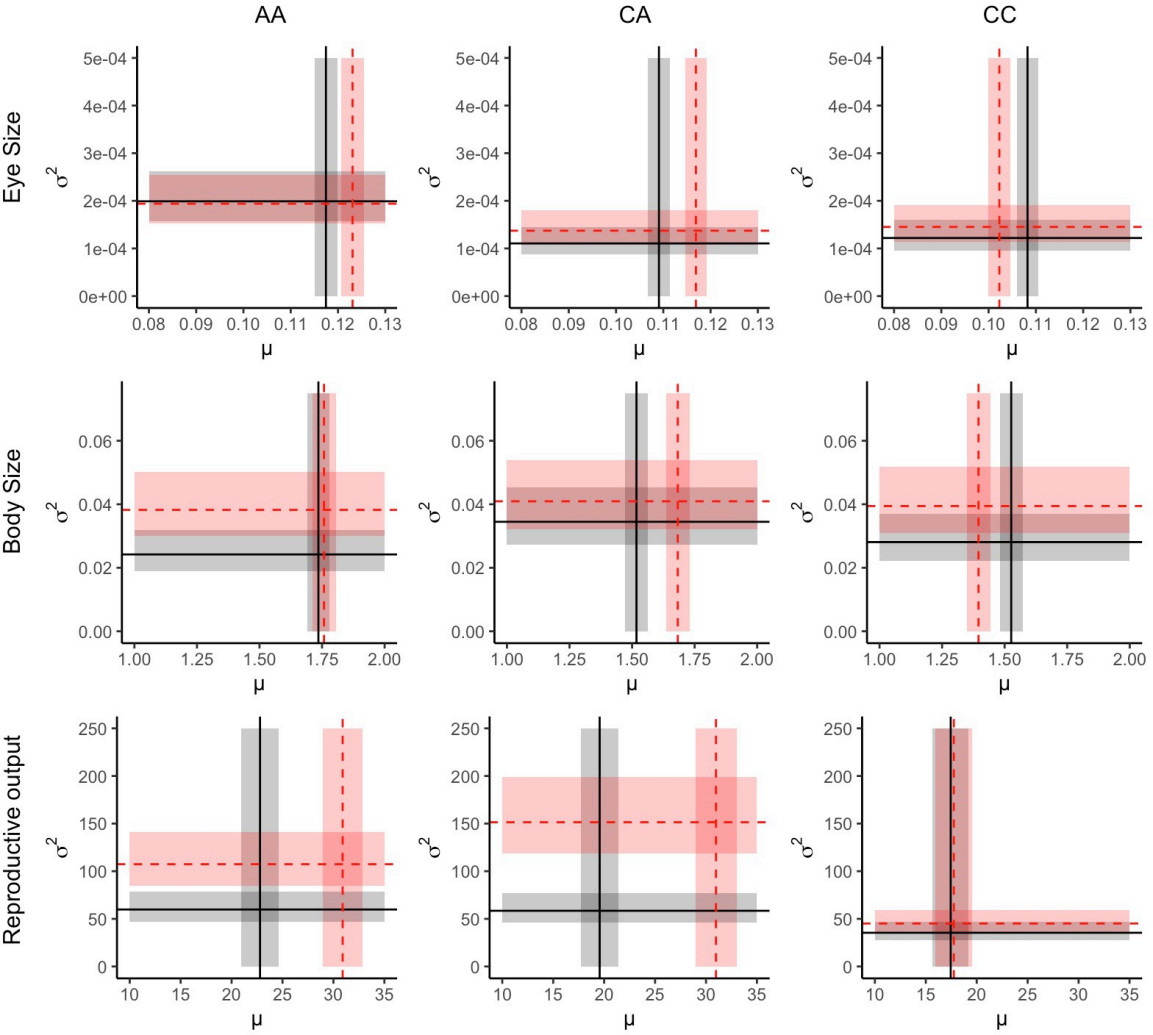
The multigenerational impacts of dietary regime on both estimated mean (*μ*) and estimated variance (*σ*^2^). Each row represents a separate trait (from top to bottom, eye size (mm), body size (mm) and reproductive output), and each column denotes a separate dietary treatment (from left to right; high-quality to high-quality, low-quality to high-quality and low-quality to low-quality). Black lines refer to generation G2 (parent) estimates, and the red dashed lines refer to generation G3 (offspring) estimates with the related shaded regions displaying the standard error for the mean and 95% confidence intervals for the variance.

We detected significant treatment-by-generation interactions for all trait means (table 2; figure 2). Constant variance was never selected as the best model. To be more specific, the variance structure differed for each trait, with variance differing by generation for body size, by treatment for eye size and an interactive impact of both treatment and generation for reproduction (electronic supplementary material, table S1).

**Table 2.** Mean trait value fixed-effect model results from linear mixed model analysis.

		*χ*	d.f.	*p*
eye size	treatment	168.165	2	<0.0001
	generation	8.4843	1	0.004
	age	31.0578	1	<0.0001
	interaction	44.7691	2	<0.0001
body size	treatment	274.1765	2	<0.0001
	generation	3.6369	1	0.05651
	age	50.7189	1	<0.0001
	interaction	70.0873	2	<0.0001
reproduction	treatment	163.973	2	<0.0001
	generation	57.993	1	<0.0001
	interaction	61.718	2	<0.0001

### (a) Eye size

Regarding eye size, the interaction in mean was driven by all but two comparisons showing significant differences from one another (table 3). The only contrasts to not show differentiation were ‘AA G2 - CA G3’ and ‘CA G2 – CC G2’. In other words, when exposed to a novel cyanobacterial food stress in G2, eye size decreases by 8.8 ± 1.5 µm. When released from such stress, eye size increases by 8 ± 2 µm in G3, i.e. to the same size as *Daphnia* exposed to algae in G2 (figure 2). Interestingly, those exposed to similar environments across generations appeared to diverge, with those continually exposed to algae increasing by 6 ± 2 µm from generation G2 to G3 and those continually exposed to cyanobacteria decreasing by the same degree (6 ± 2 µm) over the same period. Not only did algae exposure exhibit a higher mean but consistent algae exposure maintained higher variance (*σ*^2^) by approximately 47.4% compared with consistent cyanobacterial exposure.

**Table 3.** Tukey’s honestly significant difference pairwise comparisons of each trait mean. The letters in the contrast column refer to the dietary treatment group with ‘AA’ denoting high-quality to high-quality, ‘CA’ indicating low-quality to high-quality and ‘CC’ denoting low-quality to low-quality. The ‘G’ followed by a number in the same column refers to the generation.

	eye size (mm)					body size (mm)					reproduction				
contrast	estimate	s.e.	d.f.	*t* ratio	*p*-value	estimate	s.e.	d.f.	*t* ratio	*p*-value	estimate	s.e.	d.f.	*t* ratio	*p*-value
AA G2 - CA G2	0.008	0.002	697	5.06	<0.0001	0.22	0.02	697	9.79	<0.0001	3.23	1.00	694	3.22	0.017
AA G2 - CC G2	0.009	0.002	697	5.44	<0.0001	0.21	0.02	697	9.30	<0.0001	5.35	0.90	694	5.94	<0.0001
AA G2 - AA G3	−0.006	0.002	697	−3.06	0.0281	−0.02	0.02	697	−0.92	0.94	−8.11	1.20	694	−6.79	<0.0001
AA G2 - CA G3	0.001	0.002	697	0.33	1	0.05	0.02	697	2.17	0.25	−8.18	1.34	694	−6.11	<0.0001
CA G2 - CC G2	0.001	0.002	697	0.55	0.99	−0.01	0.02	697	−0.38	1	2.11	0.90	694	2.35	0.18
CA G2 - CA G3	−0.008	0.001	697	−5.36	<0.0001	−0.17	0.02	697	−6.80	<0.0001	−11.42	1.34	694	−8.51	<0.0001
CC G2 - CC G3	0.006	0.002	697	3.71	0.003	0.13	0.03	697	5.12	<0.0001	−0.31	0.83	694	−0.37	1
AA G3 - CA G3	0.006	0.002	697	3.72	0.003	0.08	0.03	697	2.87	0.048	−0.07	1.49	694	−0.05	1
AA G3 - CC G3	0.021	0.002	697	12.45	<0.0001	0.36	0.03	697	14.00	<0.0001	13.15	1.14	694	11.50	<0.0001
CA G3 - CC G3	0.015	0.002	697	9.70	<0.0001	0.29	0.03	697	10.90	<0.0001	13.22	1.30	694	10.21	<0.0001

### (b) Body size

Body size broadly followed similar patterns to eye size with the notable exception that body size was consistent in the AA treatment over both generations (table 3). *Daphnia* exposed to cyanobacteria were on average 12% smaller than their algae-exposed counterparts in generation 2 (figure 2). When released from cyanobacterial dietary stress in generation 3, they are 0.17 ± 0.02 mm larger than the previous generation, and due to potentially compounding negative effects of cyanobacteria, they are 0.29 ± 0.03 mm larger than contemporaries still exposed to cyanobacteria in generation 3. Although ‘CA 3’ grow to similar sizes as those never exposed to cyanobacteria (electroic supplementary material, figure S2), the almost negligible increase in body size across generations when consistently fed algae lead to minor but significant differences between ‘AA 3’ and ‘CA 3’ (table 3). Despite the treatments having significant effects on the mean of body size, variance was only impacted by the generation with a 36.7% increase from generation 2 to generation 3.

### (c) Reproduction

Reproductive output for all treatments exposed to algae averaged 28.2 ± 1.9 s.e. offspring across the first three clutches, whereas those groups exposed to cyanobacteria produced 18.3 ± 1.8 s.e. offspring, not considering generation. The only group not to increase reproductive output across generations was those exposed to cyanobacteria for both generations. The group that differed in dietary regime between generations (‘CA’) showed consistency with the treatment group of each generation, i.e. in generation 2, they showed no significant difference in reproduction with ‘CC 2’, and in generation 3, they showed no significant difference with ‘AA 3’. This indicates that reproduction is strongly affected by current conditions yet suffers no lasting effects of maternal condition, unlike the measured morphological traits. Reproduction also exhibited a more complex variance structure as although those exposed to algae averaged higher variation than those exposed to cyanobacteria (*σ*^2^ = 106.19, 95% CI: 83.48−139.66; *σ*^2^ = 46.31, 95% CI: 36.38−60.96 respectively), those that were exposed to algae in generation 3 but had mothers that were exposed to cyanobacteria in generation 2 had the greatest change in variance (*σ*^2^; 259% increase) between generations. In contrast those were exposed to cyanobacteria for both generations showed the lowest change (27.4% increase).

## 4. Discussion

Our results reveal that dietary stress drives both within and between generation plastic responses in *Daphnia*, underscoring the complex interplay between current environmental conditions and ancestral conditions in determining offspring phenotypes [3,40]. Across eye size, body size and reproduction, we found consistent treatment-by-generation interactions in mean trait value, but their manifestations differed fundamentally—both in direction and in the traits’ sensitivity to maternal versus current conditions. These trait-specific patterns are further supported by differences in broad-sense heritability (electronic supplementary material. figure S3), with body size exhibiting the highest heritability (*H*² = 0.29), followed by eye size (*H*² = 0.19) and finally, reproductive output (*H*² = 0.17). Notably, reproductive output also showed the largest genotype-by-environment interaction component (*H*² G × E = 0.15), suggesting greater environmental sensitivity relative to morphological traits. Given the relatively low estimates of heritability, further measurements of morphological traits—although logistically demanding—could reveal the potential for nonlinear reaction norms, which may subsequently improve such estimates [56]. Such additional resolution may also clarify the observed distinct patterns of trait-specific variance, particularly in light of not detecting an overarching mechanism of intergenerational transfer of variation, as hypothesized. Instead, our current results suggest condition transfer effects or possibly bet-hedging strategies (electronic supplementary material, table S2) are the most likely mechanisms influencing trait mean and variance under dietary stress.

For the morphological traits (eye size and body size), exposure to a low-quality food reduces average size, and with both traits, continued exposure further reduces that trait value. This is consistent with other studies that demonstrate constrained development under poor food quality [47,57]. The subsequent recovery of both traits when returned to an algal diet indicates reversible plasticity [58,59]; however, the slightly lower mean values highlight that this is not fully compensatory and is likely an effect of maternal provisioning [48], i.e. a condition transfer effect. This is partially supported by the increase in eye mean size under a high-quality diet for two generations; however, the trend for body size was weaker (table 3). Regarding the variance, eye size variation was determined by the dietary treatment and the body size variation being determined by generation. Dietary stress appears to reduce the variance in eye size, in line with predictions for within-generation plasticity [20]; however, the release of dietary stress does not promote a return to similar levels of variance, despite an increase in the mean. This is predicted by variance being in part controlled by the condition of the mother, be that either as a condition transfer effect or by producing a few well-provisioned and similar offspring, similar to a ‘conservative’ bet-hedging strategy [30,31]. Body size shows an unexpected pattern, in that generation G3 has greater variance than generation G2, irrespective of treatment. The fact that the variance pattern differs from eye size—measured simultaneously—suggests a biological rather than artefactual explanation, such as measurement error or observer bias. Although not statistically distinct (electronic supplementary material, table S3), the pattern of greatest variance differences being in the maternally matched environments (‘AA’ & ‘CC’) may suggest that body size is a useful candidate for investigations of anticipatory effects (figure 2). Many other studies have already alighted upon body size as one such candidate trait from a mean perspective [60–62], but given the decoupling of mean and variance, we suggest a potential for false negatives when only considering the mean. This could equally be considered a good example of the synergistic effects of both anticipatory effects and bet-hedging as strategies to maintain variance [32,33], as under the three environments we see a variance increase with no mean changes, a mean increase with no variance changes and a mean decrease with no variance changes within a single trait. It should be stated that simply looking at phenotypic variance alone is not sufficient to detect formal bet-hedging strategies, as those must also be accompanied with quantification of both arithmetic and geometric fitness [6]. Instead, we wish to highlight a possible mechanism for further investigation as to the adaptive value of increased body size variance.

Reproductive output showed the same treatment by interaction in mean trait differences as the morphological traits. Variance, however, differed from the morphological traits as we observed an interaction between treatment and generation. At first glance, the mean trait values suggest that current environmental conditions are the predominant drivers of phenotypic variation, as predicted by the accuracy of the experienced environmental cues [63] and partially supported by meta-analyses [9,17,26], which demonstrate rapid reversible plasticity across generations. In particular, the mean difference between generations 2 and 3 under ‘CA’ treatment regime exemplifies a lack of negative carry-over effects, counter to hypothesized condition transfer effects. Interestingly, however, the highest variance occurred in ‘CA’ individuals—those released from maternal stress—which would suggest either the potential for a diversified bet-hedging strategy or simply that a higher mean yields higher variance (as dictated by a Poisson distribution [52]). If the latter was the overriding mechanism for variance changes, then those in ‘AA’, which have the same mean in generation 3, should have a similar level of phenotypic variation. Under a Bayesian comparison, the posterior probability that the variance of ‘AA 3’ is smaller than ‘CA 3’ is 96.7% (posterior mean ratio *σ*²_AA 3_/*σ*²_CA 3_ < 1, 95% credible interval: 0.49−1.02), providing moderate evidence that there is a disconnect between mean and variance responses. The lowest variance was detected in the CC treatment group, which shows a suppressed response in both generations possibly reflecting the energetic trade-off of the current environment outweighs any maternal transfer mechanisms.

Taken together, our results demonstrate that variance and mean responses to dietary stress are often decoupled, both within and across generations. Such divergence could reveal phenotypic modulation strategies that may otherwise be overlooked, if focusing solely on central tendency measures. By implementing a simple conceptual framework, we created a series of predictions regarding the cross-generational transfer of phenotypic variation, distinguishing among condition transfer, anticipatory plasticity and bet hedging strategies. Although no single mechanism fully explained the observed results, the framework offered a valuable lens through which to interpret trait-specific responses and identify potential co-occurring strategies. Multiple strategies may also occur due to the specific form of stress, in this case, dietary stress. Cyanobacteria is widely recognized to be a poor food source for *Daphnia* species [37,64], but the exact mechanism reducing trait values remains unclear. Multiple non-mutually exclusive reasons exist, such as mechanical interference with feeding apparatus, toxicity or nutritional deficiencies [44]. While we can reject toxicity as a specific driver in this case, we cannot disentangle the role of opposing or synergistic effects between the distinct morphologies or nutritional qualities of the diets with regard to their intergenerational consequences. The possibility that nutrient content versus ingestion limitation may hint at the idiosyncratic responses observed.

As it stands, the dichotomous ‘high-’ and ‘low-quality’ diets employed support the notion that plasticity, condition transfer and bet hedging strategies are not mutually exclusive, but share an adaptive landscape shaped by trait function and environmental heterogeneity [15,32,33]. However, further attempts to predict trait mean and variance should take care to isolate individual mechanistic stressors or limitations. Alternatively, extensions to this framework may yield more accurate predictions of trait variance effects, through the incorporation of co-occurring mechanisms, greater generational depth, fine-grain environmental variation or by correlating fitness consequences of trait variance and by applying the framework to sexually reproducing organisms. In so doing, future studies can refine predictions of trait diversification or canalization under stress and in turn the evolutionary potential, a core aim of evolutionary biology.

## Data Availability

Data and code used in this study are available from the Dryad Digital Repository [65], along with the description of the data. Supplementary material is available online [66].

## References

[1] Via S, Lande R. 1985 Genotype-environment interaction and the evolution of phenotypic plasticity. Evolution **39**, 505–522. (10.1111/j.1558-5646.1985.tb00391.x) 28561964

[2] West-Eberhard MJ. 2003 Developmental plasticity and evolution. New York, NY: Oxford University Press. (10.1093/oso/9780195122343.003.0008)

[3] Weider LJ, Makino W, Acharya K, Glenn KL, Kyle M, Urabe J, Elser JJ. 2005 Genotype × environment interactions, stoichiometric food quality effects, and clonal coexistence in Daphnia pulex. Oecologia **143**, 537–547. (10.1007/s00442-005-0003-x) 15791427

[4] Vogt G. 2015 Stochastic developmental variation, an epigenetic source of phenotypic diversity with far-reaching biological consequences. J. Biosci. **40**, 159–204. (10.1007/s12038-015-9506-8) 25740150

[5] Agrawal AA, Laforsch C, Tollrian R. 1999 Transgenerational induction of defences in animals and plants. Nature **401**, 60–63. (10.1038/43425)

[6] Childs DZ, Metcalf CJE, Rees M. 2010 Evolutionary bet-hedging in the real world: empirical evidence and challenges revealed by plants. Proc. R. Soc. B **277**, 3055–3064. (10.1098/rspb.2010.0707) PMC2982066 20573624

[7] McEntire KD, Gage M, Gawne R, Hadfield MG, Hulshof C, Johnson MA, Levesque DL, Segura J, Pinter-Wollman N. 2022 Understanding drivers of variation and predicting variability across levels of biological organization. Integr. Comp. Biol. **61**, 2119–2131. (10.1093/icb/icab160) 34259842

[8] Cleasby IR, Nakagawa S, Schielzeth H. 2015 Quantifying the predictability of behaviour: statistical approaches for the study of between‐individual variation in the within‐individual variance. Methods Ecol. Evol. **6**, 27–37. (10.1111/2041-210x.12281)

[9] O’Dea RE, Lagisz M, Hendry AP, Nakagawa S. 2019 Developmental temperature affects phenotypic means and variability: a meta‐analysis of fish data. Fish Fish. **20**, 1005–1022. (10.1111/faf.12394)

[10] Gibert JP, DeLong JP. 2017 Phenotypic variation explains food web structural patterns. Proc. Natl Acad. Sci. USA **114**, 11187–11192. (10.1073/pnas.1703864114) 28973955 PMC5651739

[11] Guindre-Parker S, Mcadam AG, van Kesteren F, Palme R, Boonstra R, Boutin S, Lane JE, Dantzer B. 2019 Individual variation in phenotypic plasticity of the stress axis. Biol. Lett. **15**, 20190260. (10.1098/rsbl.2019.0260) 31337294 PMC6685001

[12] Ayroles JF, Buchanan SM, O’Leary C, Skutt-Kakaria K, Grenier JK, Clark AG, Hartl DL, de Bivort BL. 2015 Behavioral idiosyncrasy reveals genetic control of phenotypic variability. Proc. Natl Acad. Sci. USA **112**, 6706–6711. (10.1073/pnas.1503830112) 25953335 PMC4450409

[13] Mulder HA, Gienapp P, Visser ME. 2016 Genetic variation in variability: phenotypic variability of fledging weight and its evolution in a songbird population. Evolution **70**, 2004–2016. (10.1111/evo.13008) 27435892

[14] Bruijning M, Metcalf CJE, Jongejans E, Ayroles JF. 2020 The evolution of variance control. Trends Ecol. Evol. **35**, 22–33. (10.1016/j.tree.2019.08.005) 31519463 PMC7482585

[15] Sanderson S, Bolnick DI, Kinnison MT, O’Dea RE, Gorné LD, Hendry AP, Gotanda KM. 2023 Contemporary changes in phenotypic variation, and the potential consequences for eco‐evolutionary dynamics. Ecol. Lett. **26**, S127–S139. (10.1111/ele.14186) 37840026

[16] Badyaev AV. 2005 Stress-induced variation in evolution: from behavioural plasticity to genetic assimilation. Proc. R. Soc. B **272**, 877–886. (10.1098/rspb.2004.3045) PMC1564094 16024341

[17] Rowiński PK, Rogell B. 2017 Environmental stress correlates with increases in both genetic and residual variances: a meta-analysis of animal studies. Evolution **71**, 1339–1351. (10.1111/evo.13201) 28186615

[18] Simons AM, Johnston MO. 1997 Developmental instability as a bet-hedging strategy. Oikos **80**, 401. (10.2307/3546608)

[19] Vogt G, Huber M, Thiemann M, van den Boogaart G, Schmitz OJ, Schubart CD. 2008 Production of different phenotypes from the same genotype in the same environment by developmental variation. J. Exp. Biol. **211**, 510–523. (10.1242/jeb.008755) 18245627

[20] Oleksiak MF, Crawford DL. 2012 The relationship between phenotypic and environmental variation: do physiological responses reduce interindividual differences? Physiol. Biochem. Zool. **85**, 572–584. (10.1086/666904) 23099455

[21] Proulx SR, Phillips PC. 2005 The opportunity for canalization and the evolution of genetic networks. Am. Nat. **165**, 147–162. (10.1086/426873) 15729647

[22] Gomez-Mestre I, Jovani R. 2013 A heuristic model on the role of plasticity in adaptive evolution: plasticity increases adaptation, population viability and genetic variation. Proc. R. Soc. B **280**, 20131869. (10.1098/rspb.2013.1869) PMC3790484 24068357

[23] Ghalambor CK, McKAY JK, Carroll SP, Reznick DN. 2007 Adaptive versus non‐adaptive phenotypic plasticity and the potential for contemporary adaptation in new environments. Funct. Ecol. **21**, 394–407. (10.1111/j.1365-2435.2007.01283.x)

[24] Galloway LF, Etterson JR. 2007 Transgenerational plasticity is adaptive in the wild. Science **318**, 1134–1136. (10.1126/science.1148766) 18006745

[25] Uller T, Nakagawa S, English S. 2013 Weak evidence for anticipatory parental effects in plants and animals. J. Evol. Biol. **26**, 2161–2170. (10.1111/jeb.12212) 23937440

[26] Yin J, Zhou M, Lin Z, Li QQ, Zhang Y. 2019 Transgenerational effects benefit offspring across diverse environments: a meta‐analysis in plants and animals. Ecol. Lett. **22**, 1976–1986. (10.1111/ele.13373) 31436014

[27] Crino OL, Bonduriansky R, Martin LB, Noble DWA. 2024 A conceptual framework for understanding stress-induced physiological and transgenerational effects on population responses to climate change. Evol. Lett. **8**, 161–171. (10.1093/evlett/qrad037) 38370553 PMC10871929

[28] Walsh MR, Christian A, Feder M, Korte M, Tran K. 2024 Are parental condition transfer effects more widespread than is currently appreciated? J. Exp. Biol. **227**. (10.1242/jeb.246094) 38449326

[29] Bonduriansky R, Crean AJ. 2018 What are parental condition‐transfer effects and how can they be detected? Methods Ecol. Evol. **9**, 450–456. (10.1111/2041-210x.12848)

[30] Starrfelt J, Kokko H. 2012 Bet‐hedging—a triple trade‐off between means, variances and correlations. Biol. Rev. **87**, 742–755. (10.1111/j.1469-185x.2012.00225.x) 22404978

[31] Haaland TR, Wright J, Ratikainen II. 2019 Bet-hedging across generations can affect the evolution of variance-sensitive strategies within generations. Proc. R. Soc. B **286**, 20192070. (10.1098/rspb.2019.2070) PMC6939271 31771482

[32] Joschinski J, Bonte D. 2020 Transgenerational plasticity and bet-hedging: a framework for reaction norm evolution. Front. Ecol. Evol. **8**, 517183. (10.3389/fevo.2020.517183)

[33] Haaland TR, Wright J, Ratikainen II. 2021 Individual reversible plasticity as a genotype‐level bet‐hedging strategy. J. Evol. Biol. **34**, 1022–1033. (10.1111/jeb.13788) 33844340

[34] Draghi JA. 2023 Bet-hedging via dispersal aids the evolution of plastic responses to unreliable cues. J. Evol. Biol. **36**, 893–905. (10.1111/jeb.14182) 37224140

[35] Walsh MR, Packer M, Beston S, Funkhouser C, Gillis M, Holmes J, Goos J. 2018 *Daphnia* as a model for eco-evolutionary dynamics. In Life histories (eds M Thiel, GA Wellborn), pp. 403–424, vol. 5. Oxford, UK: Oxford University Press. (10.1093/oso/9780190620271.003.0016)

[36] Harney E, Paterson S, Plaistow SJ. 2017 Offspring development and life‐history variation in a water flea depends upon clone‐specific integration of genetic, non‐genetic and environmental cues. Funct. Ecol. **31**, 1996–2007. (10.1111/1365-2435.12887)

[37] Gustafsson S, Rengefors K, Hansson LA. 2005 Increased consumer fitness following transfer of toxin tolerance to offspring via maternal effects. Ecology **86**, 2561–2567. (10.1890/04-1710)

[38] Walsh MR, Whittington D, Funkhouser C. 2014 Thermal transgenerational plasticity in natural populations of Daphnia. Integr. Comp. Biol. **54**, 822–829. (10.1093/icb/icu078) 24948139

[39] Feiner N, Radersma R, Vasquez L, Ringnér M, Nystedt B, Raine A, Tobi EW, Heijmans BT, Uller T. 2022 Environmentally induced DNA methylation is inherited across generations in an aquatic keystone species. iScience **25**, 104303. (10.1016/j.isci.2022.104303) 35573201 PMC9097707

[40] Sha Y, Hansson L. 2022 Ancestral environment determines the current reaction to ultraviolet radiation in Daphnia magna. Evolution **76**, 1821–1835. (10.1111/evo.14555) 35788927 PMC9542806

[41] Sha Y, Zhang H, Wang H, Hansson LA, Niu C. 2025 Neonicotinoid insecticide causes multigenerational impairment of inducible antipredator defenses in Daphnia. Environ. Res. **271**, 121076. (10.1016/j.envres.2025.121076) 39922265

[42] Vanoverbeke J, De Meester L. 2009 Within season short-term hatching delays suggest risk-spreading behaviour in populations of the freshwater cladoceran Daphnia. Écoscience **16**, 441–451. (10.2980/16-4-3254)

[43] Gerber N, I Booksmythe, Kokko H. 2018 Sex allocation theory for facultatively sexual organisms inhabiting seasonal environments: the importance of bet hedging. Am. Nat. **192**, 155–170. 30016165 10.1086/697727

[44] Martin-Creuzburg D, von Elert E, Hoffmann KH. 2008 Nutritional constraints at the cyanobacteria–Daphnia magna interface: the role of sterols. Limnol. Oceanogr. **53**, 456–468. (10.4319/lo.2008.53.2.0456)

[45] Sarnelle O, Gustafsson S, Hansson LA. 2010 Effects of cyanobacteria on fitness components of the herbivore Daphnia. J. Plankton Res. **32**, 471–477. (10.1093/plankt/fbp151)

[46] Wejnerowski L, Cerbin S, Dziuba MK. 2017 Setae thickening in Daphnia magna alleviates the food stress caused by the filamentous cyanobacteria. Aquat. Ecol. **51**, 485–498. (10.1007/s10452-017-9631-6)

[47] Gillis MK, Walsh MR. 2019 Individual variation in plasticity dulls transgenerational responses to stress. Funct. Ecol. **33**, 1993–2002. (10.1111/1365-2435.13409)

[48] Moore MP, Whiteman HH, Martin RA. 2019 A mother’s legacy: the strength of maternal effects in animal populations. Ecol. Lett. **22**, 1620–1628. (10.1111/ele.13351) 31353805

[49] Anwer H *et al*. 2022 The effects of an obesogenic diet on behavior and cognition in zebrafish (Danio rerio): trait average, variability, repeatability, and behavioral syndromes. Ecol. Evol. **12**, e9511. (10.1002/ece3.9511) 36407899 PMC9666915

[50] Beyer JE, Hambright KD. 2017 Maternal effects are no match for stressful conditions: a test of the maternal match hypothesis in a common zooplankter. Funct. Ecol. **31**, 1933–1940. (10.1111/1365-2435.12901)

[51] Lind MI, Zwoinska MK, Andersson J, Carlsson H, Krieg T, Larva T, Maklakov AA. 2020 Environmental variation mediates the evolution of anticipatory parental effects. Evol. Lett. **4**, 371–381. (10.1002/evl3.177) 32774885 PMC7403678

[52] Nakagawa S, Schielzeth H. 2012 The mean strikes back: mean–variance relationships and heteroscedasticity. Trends Ecol. Evol. **27**, 474–475. (10.1016/j.tree.2012.04.003) 22578987

[53] Schneider CA, Rasband WS, Eliceiri KW. 2012 NIH image to imageJ: 25 years of image analysis. Nat. Methods **9**, 671–675. (10.1038/nmeth.2089) 22930834 PMC5554542

[54] R Core Team. 2024 R: a language and environment for statistical computing. Vienna, Austria: R Foundation for Statistical Computing. See https://www.R-project.org/.

[55] Pinheiro J, Bates D, R Core Team. 2025 nlme: Linear and nonlinear mixed effects models, R package version 3.1-168. See https://CRAN.R-project.org/package=nlme.

[56] Plaistow SJ, Brunner FS, O’Connor M. 2022 Quantifying population and clone-specific non-linear reaction norms to food gradients in Daphnia magna. Front. Ecol. Evol. **10**, 982697. (10.3389/fevo.2022.982697)

[57] Walsh MR, Gillis MK. 2021 Transgenerational plasticity in the eye size of Daphnia. Biol. Lett. **17**, 20210143. (10.1098/rsbl.2021.0143) 34129799 PMC8205523

[58] Relyea RA. 2003 Predators come and predators go: the reversibility of predator-induced traits. Ecology **84**, 1840–1848. (10.1890/0012-9658(2003)084[1840:pcapgt]2.0.co;2)

[59] Gabriel W. 2005 How stress selects for reversible phenotypic plasticity. J. Evol. Biol. **18**, 873–883. (10.1111/j.1420-9101.2005.00959.x) 16033559

[60] Naguib M, Gil D. 2005 Transgenerational effects on body size caused by early developmental stress in zebra finches. Biol. Lett. **1**, 95–97. (10.1098/rsbl.2004.0277) 17148137 PMC1629067

[61] Valtonen TM, Kangassalo K, Pölkki M, Rantala MJ. 2012 transgenerational effects of parental larval diet on offspring development time, adult body size and pathogen resistance in Drosophila melanogaster. PLoS One **7**, e31611. (10.1371/journal.pone.0031611) 22359607 PMC3281084

[62] Goos JM, Swain CJ, Munch SB, Walsh MR. 2019 Maternal diet and age alter direct and indirect relationships between life‐history traits across multiple generations. Funct. Ecol. **33**, 491–502. (10.1111/1365-2435.13258)

[63] Auge GA, Leverett LD, Edwards BR, Donohue K. 2017 Adjusting phenotypes via within‐ and across‐generational plasticity. New Phytol. **216**, 343–349. (10.1111/nph.14495) 28262950

[64] Wilson AE, Sarnelle O, Tillmanns AR. 2006 Effects of cyanobacterial toxicity and morphology on the population growth of freshwater zooplankton: meta‐analyses of laboratory experiments. Limnol. Oceanogr. **51**, 1915–1924. (10.4319/lo.2006.51.4.1915)

[65] Lee M, Tran KT, Castillo Y, Walsh MR. 2025 Data from: Intergenerational effects of dietary changes on trait means and variance. Dryad Digital Repository. (10.5061/dryad.q573n5tvw)40987337

[66] Lee MA, Tran K, Castillo Y, Walsh M. 2025 Supplementary material from: Intergenerational effects of dietary changes on trait means and variance. Figshare. (10.6084/m9.figshare.c.8042315)40987337

